# Reactivation of hepatitis C virus caused by steroid monotherapy for sudden deafness

**DOI:** 10.1007/s12328-024-01944-9

**Published:** 2024-04-08

**Authors:** Hiroki Kaneko, Yoshinori Ozono, Hisayoshi Iwakiri, Hiroshi Hatada, Naomi Uchiyama, Yuri Komaki, Kenichi Nakamura, Satoru Hasuike, Kenji Nagata, Hiroshi Kawakami

**Affiliations:** Division of Gastroenterology and Hepatology, Department of Internal Medicine, Faculty of Medicine, University of Miyazaki, 5200 Kihara, Kiyotake, Miyazaki, 889-1692 Japan

**Keywords:** HCV reactivation, Steroid, Sudden deafness

## Abstract

Hepatitis C virus (HCV) reactivation has been reported to be caused due to several anticancer drugs and immunosuppressive agents; however, HCV reactivation after steroid monotherapy has rarely been reported. Here, we report the case of a 65-year-old Japanese man with HCV infection who developed HCV reactivation after the administration of prednisolone (PSL) for 6 days for sudden deafness. In the patient history, the positivity for anti-HCV antibody was observed, but serum level of HCV RNA was not measured. Two months after PSL administration, the patient experienced an alanine aminotransferase (ALT) flare and the serum level of HCV RNA was observed to be 6.2 log IU/mL; then, the patient was admitted to our hospital for hepatitis treatment. Based on the clinical course and laboratory findings, the patient was diagnosed with HCV reactivation. Although the ALT levels decreased spontaneously during follow-up, they did not drop to normal range; subsequently, sofosbuvir and ledipasvir treatments were started. A sustained virological response 24 weeks after the end of treatment was achieved. This case study suggests that HCV reactivation with hepatitis flare can occur even after a steroid monotherapy, and doctors should pay attention to HCV reactivation when administering PSL for patients with HCV infection.

## Introduction

Hepatitis C virus (HCV) infection is an important public health issue and is known to be a major cause of cirrhosis and hepatocellular carcinoma [1, 2]. Recent advancements in direct acting antivirals (DAAs) have made it possible to eliminate HCV in a majority of patients, even if the patients are elderly and have comorbidities [3–5].

It is widely acknowledged that the reactivation of the hepatitis B virus (HBV) occurs subsequent to the administration of anticancer drugs or immunosuppressive agents [6–8]. At times, the reactivation of HBV proves to be fatal, prompting guidelines for the management of chronic hepatitis B to encompass recommendations aimed at averting HBV reactivation [9, 10]. In contrast, severe hepatitis is an infrequent consequence of HCV reactivation, and typically receives minimal consideration. Consequently, there remains a lack of comprehensive understanding regarding the prevalence and underlying mechanisms of HCV reactivation [11].

HCV reactivation is typically characterized by a three-fold or higher increase in the serum alanine aminotransferase (ALT) concentration among individuals lacking liver tumor infiltration, devoid of prior exposure to hepatotoxic medications, and without recent occurrences of blood transfusions or other systemic infections in addition to HCV. Changes in the levels of liver enzymes may potentially be accompanied by the reappearance of HCV RNA and/or a sudden increase in the serum level of HCV RNA ≥ 1 log IU/mL [12]. There is a paucity of reports delineating the occurrence and consequence of HCV reactivation. Here, we report the case of a patient with HCV reactivation after steroid monotherapy for sudden deafness. He developed a severe hepatitis flare, but was successfully treated with DAAs.

## Case report

A 65-year-old Japanese man was referred to our hospital owing to liver dysfunction in July 2022. In the patient history, the positivity for anti-HCV antibody more than 10 years before admission was mentioned, but serum level of HCV RNA was not measured because the ALT level remained within the normal range. He has no history of blood transfusion, tattoos, or travel abroad that could be a source of HCV infection. Additionally, his family history of HCV is unknown.

In April 2022, he was diagnosed with sudden deafness at the otorhinolaryngology department and received 30 mg/day of prednisolone (PSL) for 6 days. After that, in June, the ALT levels increased (1008 U/L) 69 days after the initiation of PSL, and serum level of HCV RNA was 6.2 log IU/mL as determined by a COBAS TaqMan HCV test (Roche Diagnostics, Tokyo, Japan). He was negative for immunoglobulin M (IgM) anti-hepatitis A antibody, IgM HBV core (HBc) antibody, anti-HBV surface (HBs) antigen, anti-HBs antibody, immunoglobulin A (IgA) anti-hepatitis E antibody, anti-nuclear antibody, and anti-liver/kidney microsome 1 antibody (Table 1). The infection patterns of Epstein–Barr virus and cytomegalovirus were previously noted. Moreover, immunoglobulin G (IgG), IgA, and IgM levels were within the normal range, and drug-induced lymphocyte stimulation test against amlodipine, previously taken orally, was negative (Table 1).

**Table 1:** Laboratory findings

Blood cell count			Biochemistry			Serological test				
WBC	4200	/μL	TP	7.3	g/dL	IgG	1431	mg/dL	ANA	Negative
Neutrophil	57.1	%	Alb	4.2	g/dL	IgA	202	mg/dL	AMA	Negative
Lymphocyte	28.5	%	T-bil	1.0	mg/dL	IgM	92	mg/dL	AMA-M2	Negative
Monocyte	8.6	%	AST	631	U/L	IgM HAVAb	Negative		ACA	Negative
Eosinophil	4.6	%	ALT	1,008	U/L	IgM HBcAb	Negative		LKM-1Ab	Negative
Basophil	1.2	%	LDH	444	U/L	HBsAg	Negative			
RBC	480 × 10^4^	/μL	ALP	211	U/L	HBsAb	Negative		**DLST**	
MCV	95.2	fl	γ-GTP	194	U/L	HBcAb	Negative		Amlodipine	Negative
MCH	30	Pg	ChE	390	U/L	HCV RNA	6.2	log IU/mL		
MCHC	31.5	%	BUN	14.7	mg/dL	HCV genotype	2A			
Hb	14.4	g/dL	Cre	1.0	mg/dL	IgA HEVAb	Negative			
Hct	45.7	%	Na	141	mmol/L	EBV VCA IgG	Positive			
Plt	26.7 × 10^4^	/μL	K	4	mmol/L	EBV VCA IgM	Negative			
			Cl	106	mmol/L	CMV IgG	Positive			
**Coagulation**			AFP	4.2	ng/mL	CMV IgM	Negative			
PT%	102	%	PIVKA-II	18	mAU/mL	M2BPGi	2.7	COI		

*WBC* white blood cell, *RBC* red blood cell, *MCV* mean corpuscular volume, *MCH* mean corpuscular hemoglobin, *MCHC* mean corpuscular hemoglobin concentration, *Hb* hemoglobin, *Hct* hematocrit, *Plt* platelets, *PT* prothorombin time, *TP* total protein, *Alb* albumin, *T-bil* total bilirubin, *AST* aspartate aminotransferase, *ALT* alanine aminotransferase, *γ-GTP* γ-glutamyl transpeptidase, *ChE* cholinesterase, *BUN* blood urea nitrogen, *Cre* creatinine, *Na* natrium, *K* kalium, *Cl* chloride, *AFP* α-fetoprotein, *PIVKA-II* protein induced by vitamin K absence or antagonist-II, *IgG* immunoglobulin G, *IgA* immunoglobulin A, *IgM* immunoglobulin M, *HAV* hepatitis A virus, *Ab* antibody, *HBc* hepatitis B core, *HBs* hepatitis B surface, *Ag* antigen, *HCV* hepatitis C virus, *HEV* hepatitis E virus, *EBV VCA* Epstein-Barr virus capsid antigen, *CMV* cytomegalovirus, *M2BPGi* mac2 binding protein glucosylation isomer, *ANA* anti-nuclear antibody, *AMA* anti-mitochondrial antibody, *AMA-M2* anti-mitochondrial M2 antibody, *ACA* anti-centromere antibody, *LKM-1* liver/kidney microsome 1, *DLST* drug-induced lymphocyte stimulation test

**Fig. 1:**
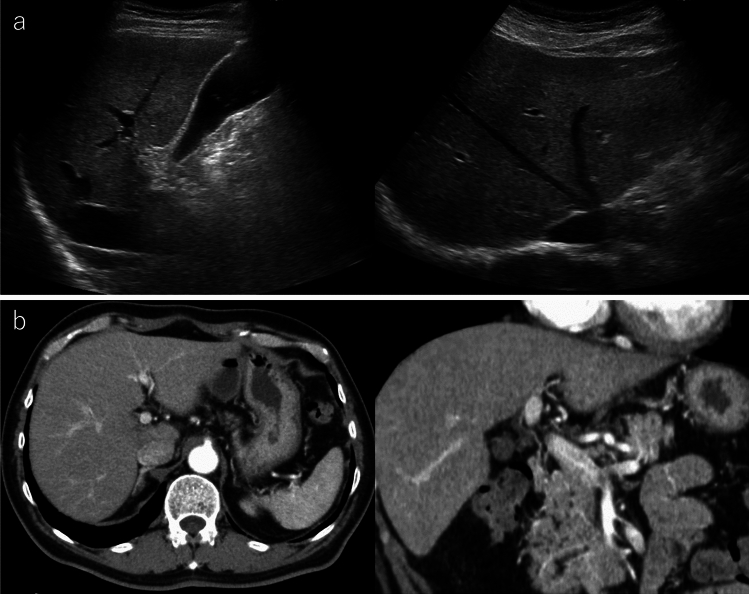
Representative images of abdominal ultrasonography and computed tomography (CT) in the present case. Images of ultrasonography (a) and CT (**b**) showing that the liver was not cirrhotic, but the liver edge was slightly dull

**Fig. 2:**
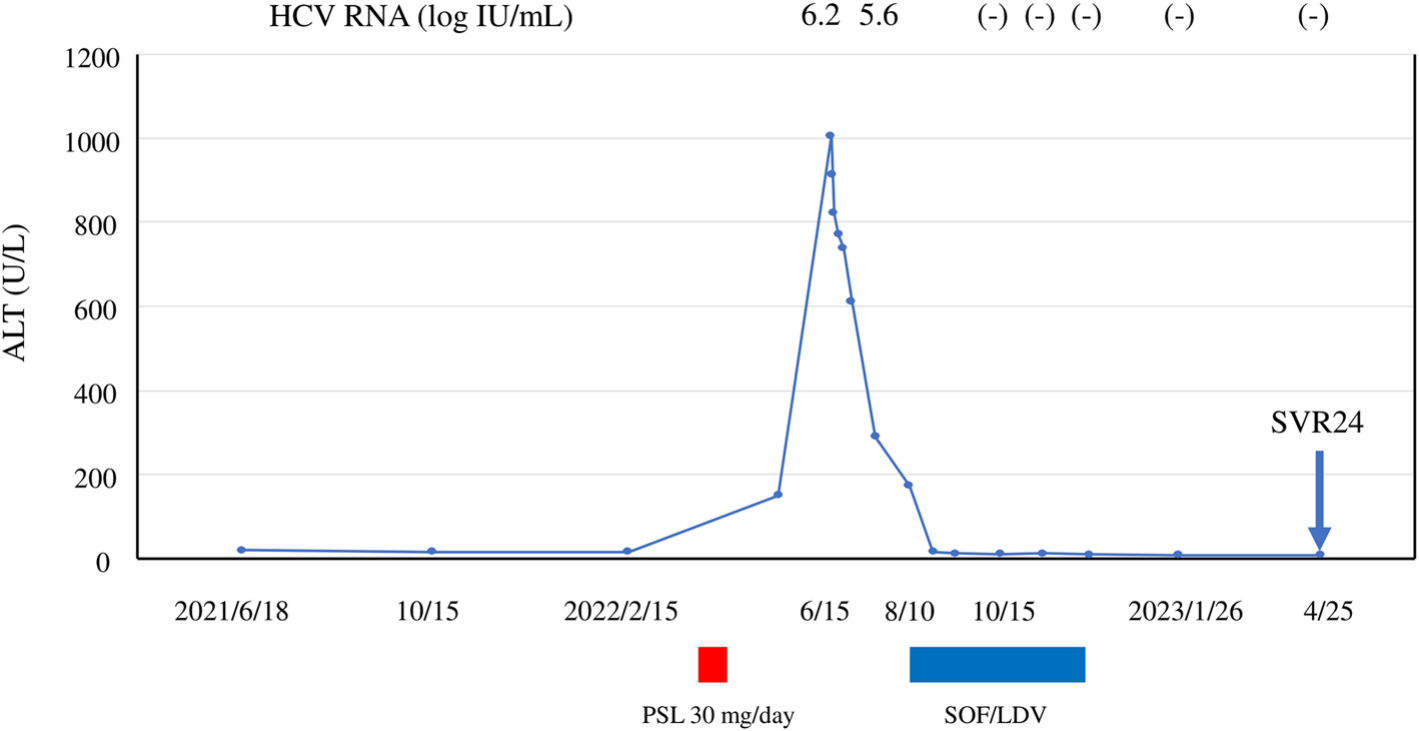
The clinical course of the patient. *HCV RNA* hepatitis C virus RNA, *ALT* alanine aminotransferase, *PSL* prednisolone, *SOF/LDV* sofosbuvir/ledipasvir

Based on the above laboratory findings, HCV reactivation was suspected; then, the patient was admitted to our hospital for hepatitis flare treatment. On first visit, the laboratory tests showed that the ALT level was 613 U/L, but total bilirubin and prothrombin time was within the normal range. Serum level of HCV RNA was 6.2 log IU/mL, and genotype was 2A. Upon additional blood tests, he was negative for anti-HBc antibody, anti-mitochondrial M2 antibody, anti-mitochondrial antibody, and anti-centromere antibody. The abdominal ultrasonography and computed tomography tests showed a pattern of chronic liver damage, and there were no ascites or tumorous regions in the liver (Fig. 1). Based on the above clinical course and laboratory findings, a diagnosis of HCV reactivation associated with PSL administration for sudden deafness was made. The patient’s clinical course was as follows (Fig. 2). Although the ALT levels decreased spontaneously during follow-up, they did not drop to normal range; therefore, sofosbuvir and ledipasvir (SOF/LDV) treatment was started for HCV infection in August. ALT was 174 U/L at the start of SOF/LDV treatment, but dropped to within the normal range after 2 weeks of treatment, and the patient completed 12 weeks of treatment without adverse effects. The serum level of HCV RNA was 5.6 log IU/mL at the start of SOF/LDV treatment, and became negative 4 weeks after the start of SOF/LDV treatment. Subsequently, sustained virological response 24 weeks (SVR24) after the end of treatment was achieved in April 2023. The patient has been followed up regularly since then, and his ALT has remained within the normal range.

## Discussion

Among cancer patients undergoing chemotherapy, liver dysfunction resulting from HBV reactivation has been observed in 14–72% of patients who did not receive prophylactic antiviral therapy, leading to liver failure in 13% of patients and death in 6% of patients. [12, 13]. On the other hand, in a prospective observational study, HCV reactivation occurred in 23% of the patients with cancer receiving chemotherapy, but none of the patients exhibited liver failure or death. [14].

HCV reactivation was reported to be caused by several anticancer drugs and immunosuppressive agents, such as rituximab, bortezomib, doxorubicin, cisplatin, docetaxel, vincristine, bendamustine, fludarabine, gemcitabine, vinorelbine, cytarabine, alemtuzumab, mitomycin, crizotinib, cyclophosphamide, methotrexate, thalidomide, fingolimod, and corticosteroids [14–19]. More recently, HCV reactivation after COVID-19 vaccination was reported [20]. Rituximab, among these agents, is reported to be a high-risk drug related to HCV reactivation [15]. In addition, steroids are likewise considered a risk factor of HCV reactivation. In most HCV reactivation cases with steroids, they were used with anticancer drugs and there are only two reports so far demonstrating HCV reactivation case caused by steroid monotherapy (Table 2) [21, 22]. In the previous reports, the peak ALT level was approximately 200–350 U/L during HCV reactivation, but in this case, the elevation was as high as 1000 U/L. In a report by Torres et al. summarizing 23 patients with cancer receiving chemotherapy and exhibiting HCV reactivation, no patients suffered from liver failure, and unlike HBV reactivation, the degree of hepatitis was often mild [14]. In addition, the total steroid dose in this case was lower, and the duration of administration was also shorter than that previously reported [21, 22].

**Table 2:** Cases of HCV reactivation caused due to steroid monotherapy

Year	Author	Age	Sex	Cirrhosis	Primary disease	HCV genotype	Diagnosis of HCV reactivation from PSL administration (weeks)	Initial dosage of PSL (mg/day)
2014	Mori [21]	76	Female	No	Dermatomyositis	2B	7	500
2021	Sato [22]	75	Female	No	Interstitial pneumonia	2A	16	30
2024	Present case	65	Male	No	Sudden deafness	2A	10	30

*HCV* hepatitis C virus, *PSL* prednisolone

In this case, serum level of HCV RNA was not measured prior to PSL administration and a liver biopsy was not performed; therefore, other possible causes of acute hepatitis, such as autoimmune hepatitis and drug-induced liver injury, cannot be completely ruled out. Most drug-induced liver injury occurs within 6 months of initiation of drug administration [23]. Given that amlodipine was initiated 3 years prior to the onset of hepatitis and the liver injury persisted after the discontinuation of amlodipine, the possibility of drug-induced liver injury due to amlodipine is extremely low. This is also supported by the fact that there was no relapse of hepatitis when amlodipine was restarted after SOF/LDV treatment for HCV. In addition, the patient has no history of taking drugs other than amlodipine, including herbal medicine and supplements. Furthermore, in this case, serologically, it was unlikely that there was any other possible cause of liver damage other than HCV, and based on the clinical course, we considered the development of a hepatitis flare as HCV reactivation. The fact that ALT remained within the normal range after achieving SVR24 may also suggest that the cause of ALT flare was HCV reactivation. The incubation period of HCV until the onset of hepatitis is 7 weeks (range 3–30 weeks) [24]. The patient has not had any blood transfusions, sexual activity, or tattoos that could cause re-infection during the period prior to the onset of hepatitis (3–30 weeks); therefore, the possibility of HCV re-infection is considered extremely low. In this case, it took approximately 2 months from the time of referral to our hospital to the start of DAA treatment. This is because SOF/LDV treatment is very expensive and it took time to receive medical subsidy for hepatitis C virus treatment in Japan.

Multiple pathogenic mechanisms have been proposed concerning the correlation between steroids and HCV reactivation. These mechanisms include an intensified infectivity of HCV resulting from an increased expression of viral receptors on the outer membrane of hepatic cells, which are recognized as pivotal players for the entry of HCV into hepatocytes [25]. In addition, steroids might induce cytokines that enhance HCV replication both in vitro and in vivo [26].

The risk of HCV reactivation with hepatitis flare is significantly associated with HCV genotype 2 [14, 27–29]. The hypervariable region 1 of the HCV envelope glycoprotein E2, which contains epitopes of neutralizing antibodies for HCV, has been known to be more variable during the course of infection in HCV genotype 2 than that in genotype 1 [27, 30]. This implies the existence of more potent antibody-mediated immune pressure on the genotype 2 virus and may help elucidate the difference in HCV dynamics observed among genotypes. The genotype of this case was type 2, similar to the two previously reported cases. In the present case, we could not obtain a serum sample before the reactivation and could not analyze the changes in nucleotides/amino acids of HCV during the reactivation. The validity of viral factors for HCV reactivation and detailed mechanisms should be elucidated in a future study.

Several studies have shown that hepatic dysfunction caused by HCV reactivation may lead to frequent alterations and interruptions in treatment for the primary disease, thereby exacerbating the overall prognosis [15, 31]. Furthermore, the long-term impact of chronic HCV infection can be severe, since the utilization of cytotoxic and immunosuppressive treatment may accelerate progression to cirrhosis [32].

In summary, we treated a rare case of HCV reactivation with severe hepatitis flare after a steroid monotherapy for sudden deafness. At present, no reliable methods exist to predict an individual’s risk of HCV reactivation, and unlike for HBV reactivation, no drugs are currently approved to prevent HCV reactivation. It should be noted that HCV reactivation can occur even if the total steroid dose is low, as in this case, or even if the dose is administered for a short period of time. The recent HCV elimination rate with DAAs therapy is extremely high and can be achieved in a short period of time even if the patients are elderly, have comorbidities, or have cirrhosis [3–5]. Then, it may be desirable to perform HCV elimination before the treatment using anticancer drugs or immunosuppressive agents for the primary disease, if possible.
